# COVID-19 and the Digitalisation of Cardiovascular Training and Education—A Review of Guiding Themes for Equitable and Effective Post-graduate Telelearning

**DOI:** 10.3389/fcvm.2021.666119

**Published:** 2021-07-02

**Authors:** Jun Hua Chong, C. Anwar A. Chahal, Ajay Gupta, Fabrizio Ricci, Mark Westwood, Francesca Pugliese, Steffen E. Petersen, Mohammed Y. Khanji

**Affiliations:** ^1^National Heart Centre Singapore, Singapore, Singapore; ^2^Cardiovascular Sciences Academic Clinical Programme, Duke-National University of Singapore Medical School, Singapore, Singapore; ^3^Lee Kong Chian School of Medicine, Nanyang Technological University, Singapore, Singapore; ^4^Department of Cardiology, Barts Heart Centre, Barts Health National Health Service Trust, London, United Kingdom; ^5^Department of Cardiovascular Diseases, Mayo Clinic, Rochester, MN, United States; ^6^Department of Cardiology, University of Pennsylvania, Philadelphia, PA, United States; ^7^NIHR Barts Biomedical Research Centre, William Harvey Research Institute, Queen Mary University London, London, United Kingdom; ^8^Department of Neuroscience, Imaging, and Clinical Sciences, Institute of Advanced Biomedical Technologies, “G.d'Annunzio” University, Chieti, Italy; ^9^Department of Clinical Sciences, Lund University, Malmö, Sweden; ^10^Casa di Cura Villa Serena, Pescara, Italy; ^11^Department of Cardiology, Newham University Hospital and Barts Heart Centre, Barts Health National Health Service Trust, London, United Kingdom

**Keywords:** COVID-19, telemedicine, education, eLearning (web-based learning/distance learning), simulation based teaching, digitalisation, diversity and inclusion, equity in access

## Abstract

The coronavirus disease-2019 (COVID-19) pandemic has had an unprecedented impact leading to novel adaptations in post-graduate medical education for cardiovascular and general internal medicine. Whilst the results of initial community COVID-19 vaccination are awaited, continuation of multimodality teaching and training that incorporates telelearning will have enduring benefit to post-graduate education and will place educational establishments in good stead to nimbly respond in future pandemic-related public health emergencies. With the rise in innovative virtual learning solutions, medical educators will have to leverage technology to develop electronic educational materials and virtual courses that facilitate adult learning. Technology-enabled virtual learning is thus a timely progression of hybrid classroom initiatives that are already adopted to varying degrees, with a need for faculty to serve as subject matter experts, to host and moderate online discussions, and to provide feedback and overall mentorship. As an extension from existing efforts, simulation-based teaching (SBT) and learning and the use of mixed reality technology should also form a greater core in the cardiovascular medicine curriculum. We highlight five foundational themes for building a successful e-learning model in cardiovascular and general post-graduate medical training: (1) digital solutions and associated infrastructure; (2) equity in access; (3) participant engagement; (4) diversity and inclusion; and (5) patient confidentiality and governance framework. With digitalisation impacting our everyday lives and now how we teach and train in medicine, these five guiding principles provide a cognitive scaffold for careful consideration of the required ecosystem in which cardiovascular and general post-graduate medical education can effectively operate. With due consideration of various e-learning options and associated infrastructure needs; and adoption of strategies for participant engagement under sound and just governance, virtual training in medicine can be effective, inclusive and equitable through the COVID-19 era and beyond.

## Background and Significance

The coronavirus disease-2019 (COVID-19) pandemic has led to significant disruption with subsequent novel adaptations identified in cardiovascular education and training (1, 2) that can be extrapolated to post-graduate medical education at large. Whilst the efficacy and safety results of community COVID-19 vaccination are awaited (3), the continuation of multimodality teaching and training that incorporates telelearning through the COVID-19 pandemic and beyond will have enduring benefit to post-graduate education as outlined below and will also place educational establishments in good stead to nimbly respond in future pandemic-related public health emergencies.

With the rise in innovative virtual learning solutions, medical educators will have to leverage technology to develop electronic educational materials that facilitate effective adult learning (4). Technology-enabled virtual learning is thus a timely progression of hybrid (combination of in-person learning with online content delivery) classroom initiatives that are already adopted to varying degrees, with a need for faculty to serve as subject matter experts, to host and moderate online discussions, and to provide feedback and overall mentorship (4). As an extension from existing efforts, simulation-based teaching (SBT) and learning should also form a greater core in the cardiovascular medicine curriculum (5). Cardiovascular medicine training programmes need a mix of what is required for medical and surgical training, incorporating essential elements that provide training in emergency/acute care scenarios, routine patient care, and also non-invasive/invasive diagnostic and therapeutic procedures (6). Other medical and surgical specialties, at both undergraduate and post-graduate levels, can thus extrapolate educational innovations developed during COVID-19 to training delivery through this pandemic and into the future (7).

Online repositories of knowledge and information that have arisen from the surge in adoption of telelearning will benefit both medical educators and learners through open resource sharing. SBT and learning have been shown to improve both technical and communication skills, which can lead to improved and safer patient care. Video encounters used in telemedicine visits can be used by educators to create learning tools for learners, and digital infrastructure associated with the rise of telemedicine can also be used in research endeavours. In addition, telemedicine solutions will benefit patient care through enabling safe distancing and making medical care more accessible to patients that cannot attend physical appointments for other reasons. The use of handheld point-of-care ultrasound will benefit patient care by providing real-time assessment of patient's physiological status and will also benefit medical educators/learners by accelerating the incorporation of ultrasound skills in the medical curriculum.

**Figure 1:**
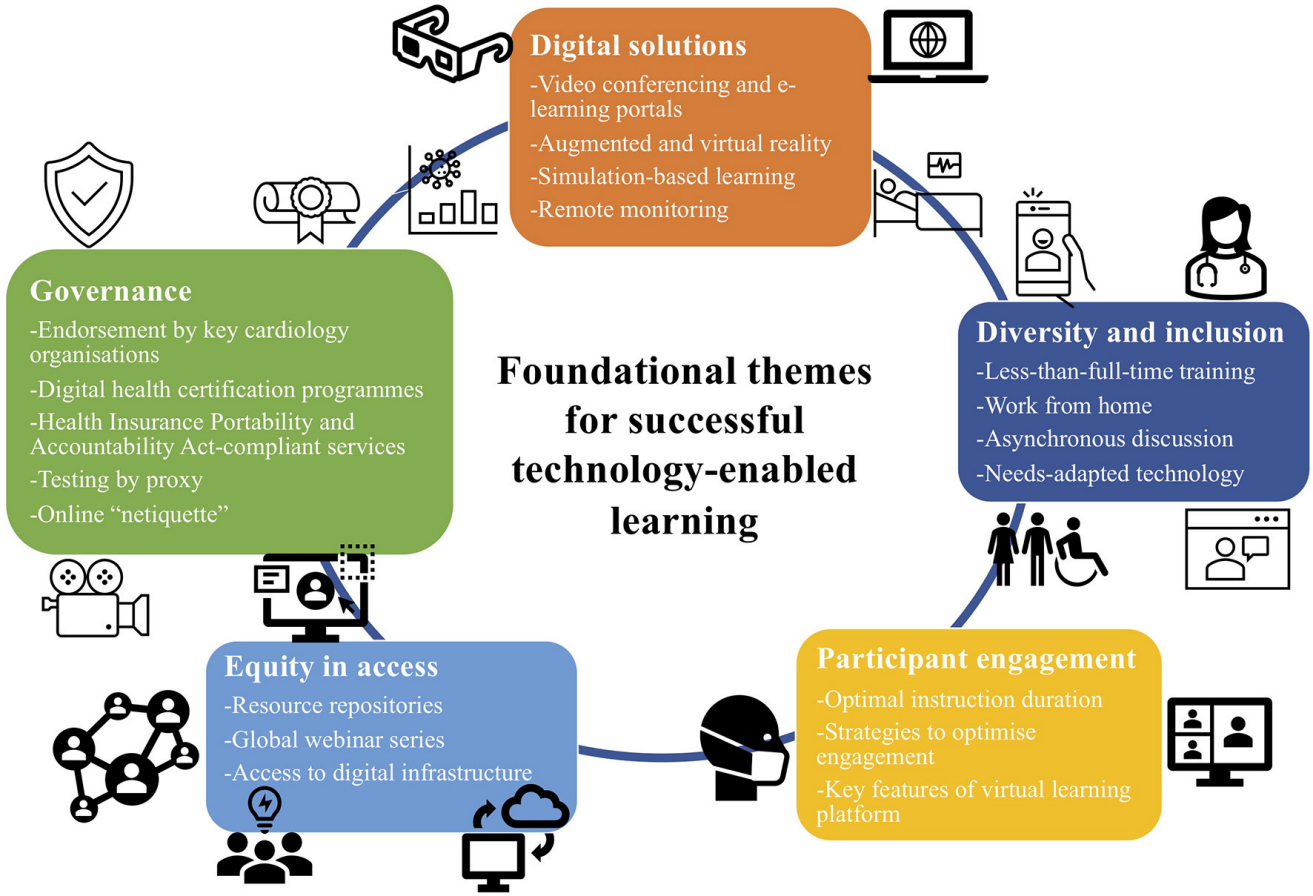
Foundational themes for building a successful e-learning model in cardiovascular and general post-graduate medical training.

Guidance on the operational aspects of conducting effective virtual teaching sessions has recently been published (8, 9). The use of technology-enabled training solutions including for research trainees have also been endorsed in various guidance papers for cardiovascular medicine trainees in different countries (2, 10). For the clinical practise of e-cardiology at large, recommendations for overcoming the challenges of digital health implementation in cardiovascular medicine have been published (11) and these concepts can also be extrapolated to enable successful digitalisation of cardiovascular training and education. We highlight and review five foundational themes for building a successful e-learning model in cardiovascular and general post-graduate medical training: (1) digital solutions and associated infrastructure; (2) equity in access; (3) participant engagement; (4) diversity and inclusion; and (5) patient confidentiality and governance framework (Figure 1). With digitalisation impacting our everyday lives, and increasingly how we teach and train in medicine, these five themes provide a cognitive scaffold for careful consideration of the required ecosystem in which cardiovascular and post-graduate medical education in general can effectively operate in the COVID-19 era and beyond.

## Digital Solutions and Associated Infrastructure

Resourceful cardiovascular medicine training programmes can experiment with digital solutions to enhance fellow education as has recently been described (12). While social distancing requirements have led to in-person lectures and national/international conference disruptions, a mainstream mechanism of distance learning and virtual meetings has rapidly evolved. Online versions of traditionally in-person conferences such as the European Society of Cardiology (ESC) Scientific Congress 2020 (13) have attracted more than three times the usual number of participants compared to previous years, a testament to overcoming the economic and travel barriers of physical distance through the use of digitalisation.

Augmented reality, extended virtual reality, mixed reality and haptic reality with various degrees of interactivity are highly applicable in educational domains (14). Problem-specific patient interviewing, review of specific disease presentations, and team training are some of the potential applications of augmented and virtual reality educational programmes.

SBT has existed for some time and can be an effective mode of procedural learning especially during these COVID-19 times. Simulator training has widely been accepted as an important tool for procedural training and is seen as an integral future development for interventional cardiology and electrophysiology training programs worldwide (6). After assessment of specific training needs, it can be used to enhance existing skills or to learn new ones. Simulation training has also been shown to facilitate acquisition of critical cardiopulmonary resuscitation skills that have the potential to impact patient outcome (15). Simulation can also help to enhance psychomotor skills, hand-eye coordination, and ambidextrous surgery, which is especially important for endoscopic surgery (16, 17). The effects of COVID-19 on procedural training can be mitigated by simulation-based e-learning delivered in a variety of formats, including the use of virtual patients, computer-based physiology simulators or advanced surgical simulators that allow learning and practise of procedures such as valve replacement surgery or coronary artery bypass grafting (CABG) (1). Simulation training has in turn been shown to translate into improved technical performance in cardiac catheterisation procedures (18, 19). High-fidelity simulation may also be used to actively engage the multidisciplinary team to enhance shared clinical knowledge and communication in emergency resuscitation situations in the cardiac catheterisation laboratory, whilst enhancing interprofessional relationships and improving patient safety (20). In advance of surgical procedures, the rendering of a computed tomography scan in an immersive virtual reality environment for reviewing anatomy and pre-operative planning of minimally-invasive CABG has also previously been described (21).

Medical trainees are required to manage complex communication scenarios effectively on entering specialty training and these scenarios include end-of-life, do-not-attempt-resuscitation and mental capacity assessment discussions. Simulation training has shown a clear benefit in amplifying the confidence and preparedness of core medical trainees in approaching these higher level communication scenarios (22).

SBT can be most valuable in the earlier stages of training or learning new procedures when there is a high propensity for complications. Trainees can control their own learning pace in a pressure-free environment with rapid feedback cycles for improved manual dexterity, whilst insulating patients from risks during this learning phase (23). Whilst SBT is useful in providing initial procedural experience in a curated environment (23), it cannot replace the educational value of in-person instruction by an experienced mentor performing procedures on an actual patient within the traditional apprentice model of medical teaching and training. In contrast, other non-invasive aspects of cardiovascular clinical training, such as cardiac imaging/electrocardiogram interpretation, may be well-suited to online delivery as each learner will have individual screen access to teaching materials which can increase learner engagement and maximise participation.

With the increasing presence of telemedicine in providing standard-of-care, video visits through a multitude of platforms will become commonplace. In this regard, interoperability of digital health services allows effective communication between two or more applications without compromise of transmitted data content (11). Interoperability enables the seamless sharing of patient health information among healthcare professionals and organisations for continuity of care in all patient contexts and across the full spectrum of caregivers and their respective clinical environments (24). When shared with preceptors, these interoperability-enabled telemedicine visits can also provide additional opportunities for feedback and self-assessment (2). Programs that use these experiences to develop their telemedicine infrastructure within a learning health system will likely reap long-term benefits in disease prevention and value-based care (25). Indeed, digital infrastructure assembled for clinical use can be multipurposed for teaching, training and even research endeavours. In the reorganisation of the heart failure unit in response to COVID-19 at an Italian hospital (26), data management staff were able to work remotely with smart-working facilities such that even during the pandemic, the team were able to collect clinical data by electronic charts and commence several retrospective and prospective trials on COVID-19 patients. Such initiatives can in turn enable trainees to keep abreast with their training research requirements and also help support trainee-initiated COVID-19 research endeavours. Deidentified multifaceted clinical data can also be assessed through big data analytics to inform future best practise and will enhance overall appreciation by trainees on how to provide cost-effective healthcare.

In recent years, new health technology sensors for physiologic variables with remote monitoring capabilities have been developed and refined (27). The necessity of using alternative care settings due to hospital bed limitations and COVID-19 infection control measures may now lead to increased uptake of innovations that make hospital-at-home care the viable preferred option for all patients except the critically ill who require intensive care. At the heart failure unit in an Italian hospital (26), patients with COVID-19 pneumonia that were stable enough to be discharged home were provided with a home-monitoring device, a T-shirt with physiological sensors able to detect heart rate, 12-lead ECG, respiratory rate and perform respiratory motion tracking (L.I.F.E. Italia) (28). The values could be remotely monitored through a server in real time. The remote assessment of various vital signs parameters can present a unique lesson for trainees in the physiology of a recovering COVID-19 patient and can be a novel way to develop clinical acumen through the interpretation of vital signs without immediate correlation with bedside physical patient assessment. In this regard, a structured tele-education critical care program using case-based learning and intensive care medicine management principles has been shown to facilitate knowledge translation and quality improvement in the critical care setting (29). Medical trainees undertaking a remote critical care rotation would still be required to learn and practise the systems-based approach in the assessment of the critically ill patient, report on patients' conditions during daily rounds, and propose a management plan for each systems-based issue (30). One limitation of undertaking a remote rotation would be lack of hands-on experience with standard intensive care medicine interventions such as central catheter placements and advanced airway support (30). Such a deficiency could be countered by remote video monitoring capabilities that allow trainees to be virtually present in patient rooms at all times, such that learners can continue to observe the conduct of intervention procedures (30). Remote monitoring of arterial lines, mechanical ventilators, dialysis machines, and infusion pumps would also allow for more accurate clinical assessment (30). Research into the effectiveness of these promising newer systems in remote intensive care medicine education and training is required before widespread implementation.

In the provision of acute cardiovascular care and investigations during the COVID-19 pandemic, hand-held point-of-care ultrasound (HH-POCUS) is particularly suited for rapid, focused, and integrated assessment of the heart and lungs that can minimise duration of direct patient contact (31). Due to rapid image availability and immediate bedside interpretation, POCUS with pocket-sized dedicated devices and, more recently, “plug-and-play” ultrasound probes that can be interfaced with mobile devices, provides rapid, “real-time” information that can immediately inform patient management (32). In the COVID-19 pandemic, there have also been calls to replace stethoscope auscultation by wireless HH-POCUS of the chest to minimise patient contact. Lung ultrasonography (LUS) requires minimal technical skills and <2min to perform. LUS can accurately diagnose respiratory interstitial syndromes, especially in critically ill and emergency medicine patients (31). The adoption of HH-POCUS in the acute cardiopulmonary assessment of COVID-19 patients will enhance acute cardiovascular care delivery and enable cardiovascular medicine trainees to hone their skills in rapid ultrasound assessment of acutely unwell patients. Indeed, there has been a push to incorporate POCUS training as integral parts of the medical education curriculum (33). In this regard, independent cardiac ultrasound learning, combined with use of e–learning software and self-practise, is feasible and performs as well as a validated, bedside, frontal cardiac ultrasound course (34).

During the COVID-19 pandemic, telehealth strategies can also be leveraged to provide remote chronic disease management. In fact, the pandemic has spurred a call for action to adopt efficacious and cost-effective cardiac telerehabilitation intervention (35). Telehealth enables the delivery of care remotely using information and communication technologies and provides a safer solution for patients and staff in the midst of COVID-19 pandemic in keeping with social distancing mandates (36). The transition to the use of bespoke telerehabilitation platforms that include remote monitoring devices, video conferencing and mobile apps will also future-proof cardiac rehabilitation as the push to digitalisation continues into the pandemic and beyond (36). Telehealth solutions can also be applied to any patient that may be unable to physically attend health services for other reasons (35). In turn, the maturation of telehealth infrastructure will enhance the virtual learning experience for trainees as they learn to correlate physiological information from remote monitoring devices with video assessment of chronic cardiac patients. With appropriate consent and governance, these video encounters can also be shared as learning tools for future trainees on how to deliver effective heart failure and cardiac rehabilitation care. Cardiac telerehabilitation has been shown to decrease morbidity and mortality as facility-based cardiac rehabilitation programs (37). Medical trainees can apply core competencies of the clinical skills curriculum such as motivational interviewing skills to encourage uptake and promote adherence to a physical activity schedule (30). Supervising members of the rehabilitation team could provide feedback on trainees' communication skills and counselling efficacy after receiving quantitative feedback from step counters (30).

Virtual learning has strengths and limitations. On one hand, spontaneous discussion and face-to-face interaction are restricted, leading to lack of grasp of a patient that comes only at the physical bedside. Previously demarcated whole-day training time for in-person learning may also be compromised through the interlace of e-learning and clinical duties, and training programmes will need to ensure protected time is made available for online professional development through adaptive teaching schedules that will maximise trainee engagement and effectiveness of content delivery. On the other hand, remote meetings provide geographic and temporal flexibility and reduce commuting and physical space requirements. The advances in technology can be helpful in discussions and content delivery from expert faculty worldwide and can also help compensate for scarcities of academic staff, including instructors, teachers and facilitators (38). E-learning is also cost-effective in that it offers learning opportunities for a large number of learners with little need for physical space (38). Education sessions can also be recorded for future repeated review, thus enabling spaced repetition which is the key to long-term knowledge reinforcement (39). Table 1 outlines the various advantages and disadvantages of e-learning as described in this paper.

**Table 1:** General pros and cons of e-teaching and e-learning.

**Pros**	**Cons**
**E-learning**	
Not restricted to a regular course day and can take place in a variety of locations with increased access and reach	Need for access to computing facilities (workstations, internet broadband, hardware resource, software applications)
Efficient learning experience and wider learning curriculum offered	Lower mental and physical readiness and hazards related to increased screen time
Increased ability to adhere to learning schedule due to flexibility in time and space	Feeling of anxiety for not learning the curriculum
Agile on-demand training	
**E-teaching**	
Safest and easiest ways to impart education in a pandemic	Lack of interaction among learners and teachers with possible focus deficit
Lectures can be recorded and shared for reference with a wide range of audiences	Difficulty in assessment of different domain progress
Affordability, no need for real estate and transportation	Limited technology infrastructure in remote areas and least developed countries
	Protection of privacy, confidentiality and security of patient health information

## Equity in Access

Global experts can be engaged for regular teaching webinars on core cardiovascular medicine curriculum topics which can then be made accessible to all (40). Consultants and anyone else interested across the globe can request access by sending a message through social media platforms or email as part of their continuing medical education. Live viewers can also request “certificates of viewing.” Webinars can be recorded and hosted in a video gallery. Recent such efforts have been very well-received with some reaching almost 500 views in <3 weeks (40). Organisational support and endorsement through professional societies can enable equitable sharing of educational resources in cardiovascular medicine education between different socioeconomic areas.

Furthering the education democratisation movement, societies such as the Association of American Medical Colleges (AAMC) has responded to the wealth of innovative ideas from faculty by creating a freely available resource repository that will allow for sharing and dissemination of collated educational approaches (41), further improving national and international access to useful resources that enable learning and collaboration.

However, overreliance on virtual instruction methods could worsen digital inequality with regards to internet access, computer skills and equipment availability, and application of digital knowledge (42). To ensure equitable access to the benefits of virtual learning, essential digital infrastructure such as internet and computer devices needs to be made accessible to learners from varying demographics.

## Participant Engagement

Technology is not without drawbacks. Some learners report mild eyestrain, headaches, or motion sickness when using virtual and mixed reality devices (43). These symptoms resolve quickly after taking off the device, but will likely limit the duration of study and how much information can be conveyed in one sitting. Within virtual reality, educators had optimal delivery periods of maximum 10-min, when compared to traditional 2-h lectures (43).

Several strategies to optimise virtual participant engagement have been highlighted (8). First, ensure all potential users are oriented to the group sharing application. Second, request the audience to place their microphones on mute unless they wish to speak to minimise unintended interruptions. Third, encourage participants to use speaker-view video to allow for a more dynamic and engaging experience. Of note, this increased engagement with video can be coupled with the ability to blur the user's background to maintain privacy. Fourth, assign a moderator who can solicit participant opinions and also give participants who have not had an opportunity to speak a chance to do so. Finally, designate someone to assist with troubleshooting technical problems that can otherwise disrupt the flow of the session (8).

The following key features of a virtual learning platform can help enable effective integration, collaboration, education and communication for optimal participant engagement (8). The chosen platform should have an integrative approach with availability as an application on desktops, phones and web browsers. Collaboration should be enabled through document/slides sharing and editing functions with ready access for fellowship administration and other colleagues. As an educational tool, the platform should enable audience polling for better engagement and quicker feedback, with live stream and record options. Targeted communication regarding virtual meetings should be sent out to the appropriate audience (e.g., first vs. second-year trainees) to reduce email burden and optimise reach.

## Diversity and Inclusion

Virtual learning platforms can provide sustainable, high-quality educational infrastructure that fosters participation and collaboration. This may be especially valuable for trainees on maternity or paternity leave and can serve as a solution for physical separation. Improved flexibility for deployment, including options of working from home, is thus an important aspect in the ability to recruit and retain physician caregivers. This may prove to be a positive addition to workforce diversification as women are more likely to carry the bulk of these responsibilities (44, 45). The core cardiovascular medicine curriculum requires competencies in multiple practical skills including coronary angiography, cardiac pacing, and echocardiography. Achieving these skills often requires working outside of scheduled hours. In contrast, regular hours and family conduciveness are important factors for consideration particularly for female trainees in deciding on their future career (44). Flexibility in training is also a pertinent consideration when planning a family and if there is a perception of a lack of support for less-than-full-time (LTFT) training in cardiovascular medicine, this may also deter potential female applicants who have competing family responsibilities. Perceptions of the rigidity of cardiovascular medicine training will need to change for women to assess that they are equally able to have a successful career as a cardiologist. In this regard, virtual solutions can help level the playing field for aspiring female cardiologists who may now avail of the opportunities more easily through remote learning and simulation-based skills training that can also complement LTFT cardiovascular medicine training.

Online learning material can be more accessible than analogue content for learners with disabilities as electronic text can be read aloud and expressed in braille form and audio content can be transcribed to text form. The e-learning environment can also provide learners with more temporal and geographic flexibility with various teaching mediums such as tutorial discussions on asynchronous communication portals. Needs-adapted technology through web-based e-learning platforms will enhance the learning experience for both cardiovascular medicine trainees with disabilities and the broader trainee population.

## Patient Confidentiality and Governance Framework

On the March 30th, 2020, the cardiovascular medicine specialist advisory committee within the Joint Royal College of Physicians Training Board in the United Kingdom, informed all cardiovascular medicine fellows that training was on pause. The “CardioWebinars during COVID-19” programme to continue education and help maintain trainee morale over this period was thus developed by cardiovascular medicine registrars and supported by the British Cardiovascular Society in recognition of its value (40). In early April, they reached out to the cardiology community on social media to help develop a programme, and received an incredible response from experts in various cardiology subspecialties volunteering to speak on their subject. They have been able to schedule three webinars a week from mid-April to early June, with topics that span the core cardiovascular medicine curriculum. Full webinar schedule is made available on their website and *via* their Twitter handle. Similar efforts in other countries can also succeed with support from key organisations of these initiatives and provide an important form of endorsement and governance. Peer-review for vetting educational material quality is essential to ensure that the highest training standards are maintained. In addition, the need to establish internationally-recognised digital health certification programmes as a form of standardised governance for clinical delivery has been identified as a challenge to overcome in digital health implementation in cardiovascular medicine (11).

Compliance to applicable digital health directives should be ensured (11). Patient confidentiality should be protected through the use of platforms like Zoom and WebEx that allow sharing of patient-level data through Health Insurance Portability and Accountability Act-compliant services (2). E-learning ground rules and code-of-conduct should be established to help ensure a fair and principled construct within which regulated virtual training is conducted (12). Strategies to perform remote examination invigilation include requesting the examination candidate to demonstrate a scan of the room for educational materials prior to starting the online examination and mandating that audio and video functions are enabled throughout the examination for continuous remote supervision. Establishment of online etiquette (“netiquette”) can help maintain effective and respectful online discussions (46).

## Conclusion

The COVID-19 pandemic has thus accelerated the digitalisation of cardiovascular medicine training, education and learning at an unprecedented pace. The maturation of technological infrastructure for acute and chronic remote cardiac care provision will also improve e-learning capabilities for trainees. With due consideration of the various technology-enabled learning solutions, associated infrastructure needs and adoption of strategies to maximise participant engagement under fair and just framework and governance, the benefits of virtual learning can be leveraged in cardiovascular medicine and wider medical education to provide effective, inclusive and equitable training for the current and future generation of doctors through the current COVID-19 era and beyond.

## Author Contributions

JC and MK contributed to the conception and design and agree to be accountable for all aspects of the work in ensuring that questions related to the accuracy or integrity of any part of the work are appropriately investigated and resolved. JC, CC, FR, and MK performed the literature review and contributed to the initial drafting of the paper. JC, CC, AG, FR, MW, FP, SP, and MK provided critical revision of the paper and provided final approval of the version to publish. All authors contributed to the article and approved the submitted version.

## Conflict of Interest

The authors declare that the research was conducted in the absence of any commercial or financial relationships that could be construed as a potential conflict of interest.
